# Hyperoxia in portal vein causes enhanced vasoconstriction in arterial vascular bed

**DOI:** 10.1038/s41598-020-77915-0

**Published:** 2020-12-01

**Authors:** Dilmurodjon Eshmuminov, Dustin Becker, Max L. Hefti, Matteo Mueller, Catherine Hagedorn, Philipp Dutkowski, Philipp Rudolf von Rohr, Maximilian Halbe, Stephan Segerer, Mark W. Tibbitt, Lucia Bautista Borrego, Martin J. Schuler, Pierre-Alain Clavien

**Affiliations:** 1 Department of Surgery, Swiss Hepato-Pancreato-Biliary and Transplantation Center, University Hospital Zurich, Zurich, Switzerland; 2 Transport Processes and Reactions Laboratory, Department of Mechanical and Process Engineering, ETH Zurich, Zurich, Switzerland; 3 Wyss Zurich ETH Zurich/University of Zurich, Sonneggstrasse 3, ML H 19, 8092 Zurich, Switzerland; 4 Department of Cardiac Surgery, University Hospital Zurich, Zurich, Switzerland; 5 Division of Nephrology, Dialysis and Transplantation, University Department of Medicine, Kantonsspital Aarau, Aarau, Switzerland; 6 Macromolecular Engineering Laboratory, Department of Mechanical and Process Engineering, ETH Zurich, Zurich, Switzerland

**Keywords:** Gastrointestinal models, Gastroenterology, Medical research, Biomedical engineering

## Abstract

Long-term perfusion of liver grafts outside of the body may enable repair of poor-quality livers that are currently declined for transplantation, mitigating the global shortage of donor livers. In current ex vivo liver perfusion protocols, hyperoxic blood (arterial blood) is commonly delivered in the portal vein (PV). We perfused porcine livers for one week and investigated the effect of and mechanisms behind hyperoxia in the PV on hepatic arterial resistance. Applying PV hyperoxia in porcine livers (n = 5, arterial PV group), we observed an increased need for vasodilator Nitroprussiat (285 ± 162 ml/week) to maintain the reference hepatic artery flow of 0.25 l/min during ex vivo perfusion. With physiologic oxygenation (venous blood) in the PV the need for vasodilator could be reduced to 41 ± 34 ml/week (p = 0.011; n = 5, venous PV group). This phenomenon has not been reported previously, owing to the fact that such experiments are not feasible practically in vivo. We investigated the mechanism of the variation in HA resistance in response to blood oxygen saturation with a focus on the release of vasoactive substances, such as Endothelin 1 (ET-1) and nitric oxide (NO), at the protein and mRNA levels. However, no difference was found between groups for ET-1 and NO release. We propose direct oxygen sensing of endothelial cells and/or increased NO break down rate with hyperoxia as possible explanations for enhanced HA resistance.

## Introduction

The unprecedented success of liver transplantation over the past three decades has restored normal function to many patients suffering from acute and end-stage liver failure. However, the proliferation of life-changing transplantation has resulted in a worldwide shortage of available liver grafts^1,2^. Thus, strategies to expand the pool of suitable organs for transplantation is a major focus of clinical research^2,3^. Ex vivo machine perfusion has emerged as a leading technology to increase the available donor pool, as this approach can rescue injured grafts, initially not suitable for transplantation, and concomitantly predict post-transplant graft function^4–6^.

The liver is a highly perfused organ, receiving 25% of the cardiac output while comprising only 2.5% of the body weight^7^. The liver possesses a dual blood supply from the portal vein (PV) and the hepatic artery (HA) as well as a unique microcirculation that mixes the arterial blood (high oxygen saturation) from the HA with the venous blood (partial oxygen saturation) from the PV. However, a recent systematic review of ex vivo liver perfusion protocols demonstrated that non-physiologic arterial blood delivery to the PV is common in both the clinical and experimental settings^8,9^. It is necessary to supply sufficient oxygen in order to maintain viability of the metabolically active liver; however, the value of preventing excessive oxygen is not as intuitive despite the fact that excessive oxygenation could be deleterious in the clinical setting^10,11^. Further, the specific effect of delivering arterial blood to the PV on liver hemodynamics has not been explored, owing to the fact that such experiments are difficult to perform in vivo.

Our multidisciplinary team, consisting of surgeons, biologists, and engineers, recently introduced a liver perfusion technology that enables preservation of injured human livers for up to one week^5^. The technology serves as an “artificial body” that recapitulates core bodily functions to maintain liver viability ex vivo. The initial machine setup was based on the state of the art^8^, and additional functionality was introduced to the perfusion machine sequentially. The biological relevance of each unit operation was demonstrated using porcine livers^5^. Based on current machine perfusion protocols, we supplied the PV with arterial oxygen content (oxygen saturation: 95–100%) during our initial experiments^8,9^. However, the blood supply of the PV in vivo is at venous oxygen saturation (< 80%) and we hypothesized that mimicking this oxygen saturation would improve liver function during machine perfusion. Therefore, we modified our perfusion protocol to supply the PV with venous blood. Surprisingly, we observed significant variation in the HA resistance depending on oxygen content in the PV^5^. In this study, we related the observed hemodynamic differences in the HA in a porcine model to the release of vasoactive substances. Our findings suggest that direct oxygen sensing of endothelial cells and/or increased NO break down rate with hyperoxia may cause enhanced HA resistance.

## Materials and methods

### Perfusion machine

**Figure 1:**
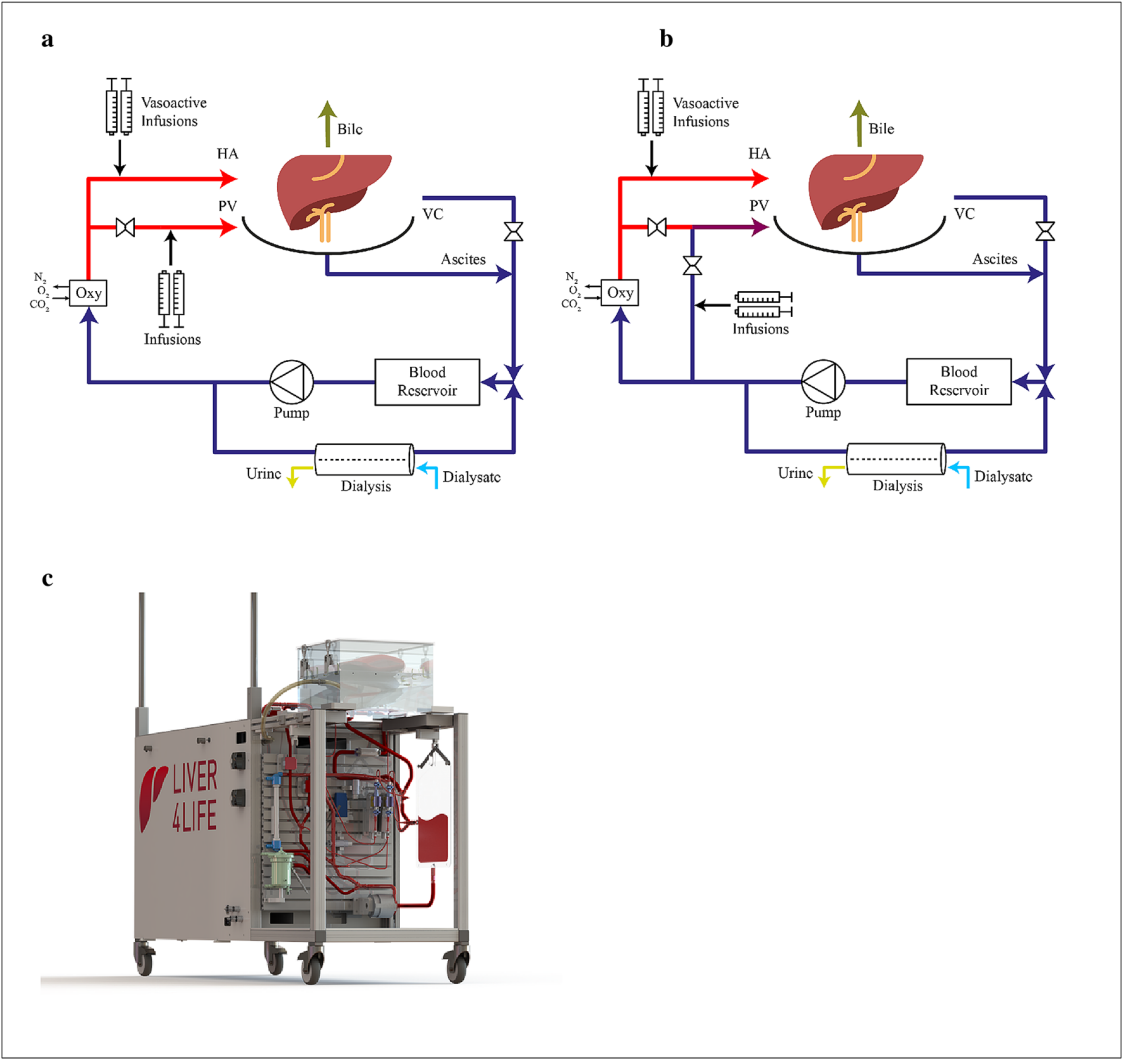
Schematic illustration of the perfusion loops for arterial PV (**a**) and venous PV groups (**b**). (**a**) In arterial PV group, arterial blood was provided in the PV with total PV flow rate of 1 l/min. For this purpose blood flow was split after the oxygenator into the PV and HA lines with the same oxygen content. (**b**) In venous PV group, venous blood was provided in the PV with total PV flow rate of 1 l/min. For this purpose, oxygen deprived blood from the liver output was mixed with oxygenated blood after oxygenator. Flow rates in the arterial and deoxygenated lines were automatically adjusted to maintain an oxygen saturation at the liver output/outlet of ~ 65%. (**c**) Representative illustration of the perfusion machine. *HA* hepatic artery, *PV* portal vein, *VC* vena cava.

The engineered liver perfusion machine includes one pump operating in pulsatile mode, an automated dialysis unit, an “artificial pancreas” based on feedback controlled insulin infusions depending on real time glucose measurements in blood, tight automated hemodynamic control, diaphragm simulation, and one oxygenator with controlled gas mixture supply (N_2_, O_2_, CO_2_) to maintain physiologic blood gas parameters (Fig. 1). For a detailed description of the development process of the perfusion machine and the applied perfusion protocol, we refer to our previous work^5^. Amino acids (for nutrition), antibiotics, bile acids, and steroids were provided continuously by means of direct infusion to the blood supply^5^. Blood gas parameters including pO_2_ (partial pressure O_2_), pCO_2_ (partial pressure CO_2_), and pH were measured in the HA and PV by online blood gas sensors (Terumo, CDI 500). Based on the current levels of pO_2_ (target 10–12 kPa) and pH in the HA, an automated algorithm defined the individual required gas flow rates of O_2_, N_2_, and CO_2_ supplied to the oxygenator. The system continuously measured the oxygen saturation in the vena cava (vSO_2_) by the Terumo, CDI 500 device.

### Oxygen content and consumption

Oxygen content (1), delivery (2), and consumption (3) were calculated by the following equations:

1. Oxygen content (C*O_2_) (mlO_2_/dL) = (1.34 × hemoglobin concentration (g/dL)  × SO_2_(%)/100%) + (0.0031 × PO_2_ (mmHg))
  * signifies the location of measurement (HA, PV, or VC). Note, online VC oxygen content was calculated without the pO_2_ value in the VC line.
2. Oxygen delivery (DO_2_) (mlO_2_/min) = (HA flow(ml/min)xC^a^O_2_) + (PV flow(ml/min)xC^pv^O_2_)
3. Oxygen consumption(VO_2_) (mlO_2_/min) = DO_2_-(HA + PV flow(ml/min))xC^vc^O_2_

### Porcine livers and experimental groups

Livers were procured respecting local animal protection regulation.

Livers and blood were procured under general anesthesia in a standardised manner^5^. The animal study protocol was approved by veterinarian office of Canton Zurich (registration number: 26538-ZH079/15). All methods were in accordance with relevant guidelines and regulations. During surgery, in vivo flow in the HA and PV was measured with VeriQ (Medistim). After back table preparation, the livers were connected to the perfusion machine primed with autologous leukocyte depleted blood at 34 °C. The targeted perfusion duration was one week. This target was based on the ability of human organs to regenerate in vivo within this time period^12,13^.

The following experimental groups were designed:

- Group 0 “Initial” (n = 4): Initial experiments were performed providing arterial blood in the PV without HA hemodynamic control. For arterial oxygen content in the PV, blood flow after the oxygenator is split into two lines: one for the PV line and the second for the HA line (Fig. 1 a). Consequently, the HA and PV had similar blood gas parameters. A pinch valve controlled the flow in the PV to 1 l/min. vSO2 in the VC line was recorded but not used for controlling any parameters. The HA pressure was maintained at mean arterial pressure (MAP) > 65 mmHg. In vivo, PV flow is not controlled by the liver and depends on intestinal blood flow regulation^7^. Thus, the PV was provided with a fixed flow rate of 1 l/min.
- Group 1 “Arterial PV” (n = 5): Arterial oxygen content was delivered in the PV as for Group 0 and the HA resistance was controlled tightly. The minimal HA flow rate was defined as 0.25 l/min. If the flow rate reduced to less than 0.25 l/min, the system injected the vasodilator Nitroprussiat (Nitropussiat Fides, Rottapharm, 1 mg/ml) in an automated manner. Note, the reference flow parameters in the HA (0.25 l/min) and PV (1 l/min) were based on in vivo flow measurement with VeriQ (Medistim) during liver procurement.
- Group 2 “Venous PV” (n = 5): Physiologic venous oxygen content was delivered in the PV. To achieve the desired oxygen saturation levels, blood from the reservoir (oxygen saturation ~ 65%) was mixed with a portion of arterial blood from the oxygenator (Fig. 1 b). The control algorithm adjusted the mixing fractions such that the VC oxygen saturation (vSO_2_), which was monitored continuously, remained at 65%. For this purpose, the system controlled the bypass flow rate from the oxygenator and reservoir individually, while maintaining a total blood flow rate of 1 l/min^5^. Similar to Group 1 (Arterial PV), HA resistance was controlled by infusions of Nitroprussiat.

### Measurements and analytics

Liver function was evaluated with adenosine triphosphate (ATP) production in tissue, bile flow, blood urea nitrogen, and lactate clearance. The following injury markers were evaluated in the perfusate: aspartate aminotransferase (AST). Cytochrome C for mitochondrial injury, 8-hydroxy-2-deoxy guanosine for DNA damage in perfusate. The mRNA expression of cytokines (IL-6, IL-10), ICAM-I (Intercellular Adhesion Molecule-1) for endothelial cells, and TLR4 for Kupffer cells were measured. Histology was performed with haematoxylin–eosin (H&E) staining for necrosis and von Willebrand factor (vWF) staining for endothelial activation^14^.

### Statistics

Matlab R2017a (MathWorks, USA) was used for statistical analysis and graphs. Data was reported as mean with standard deviation (s.d.) with p value < 0.05 as significant. Two-tailed Student’s t-test was used to compare study groups.

## Results

### Tight hemodynamic control was necessary to reach one week perfusion

**Figure 2:**
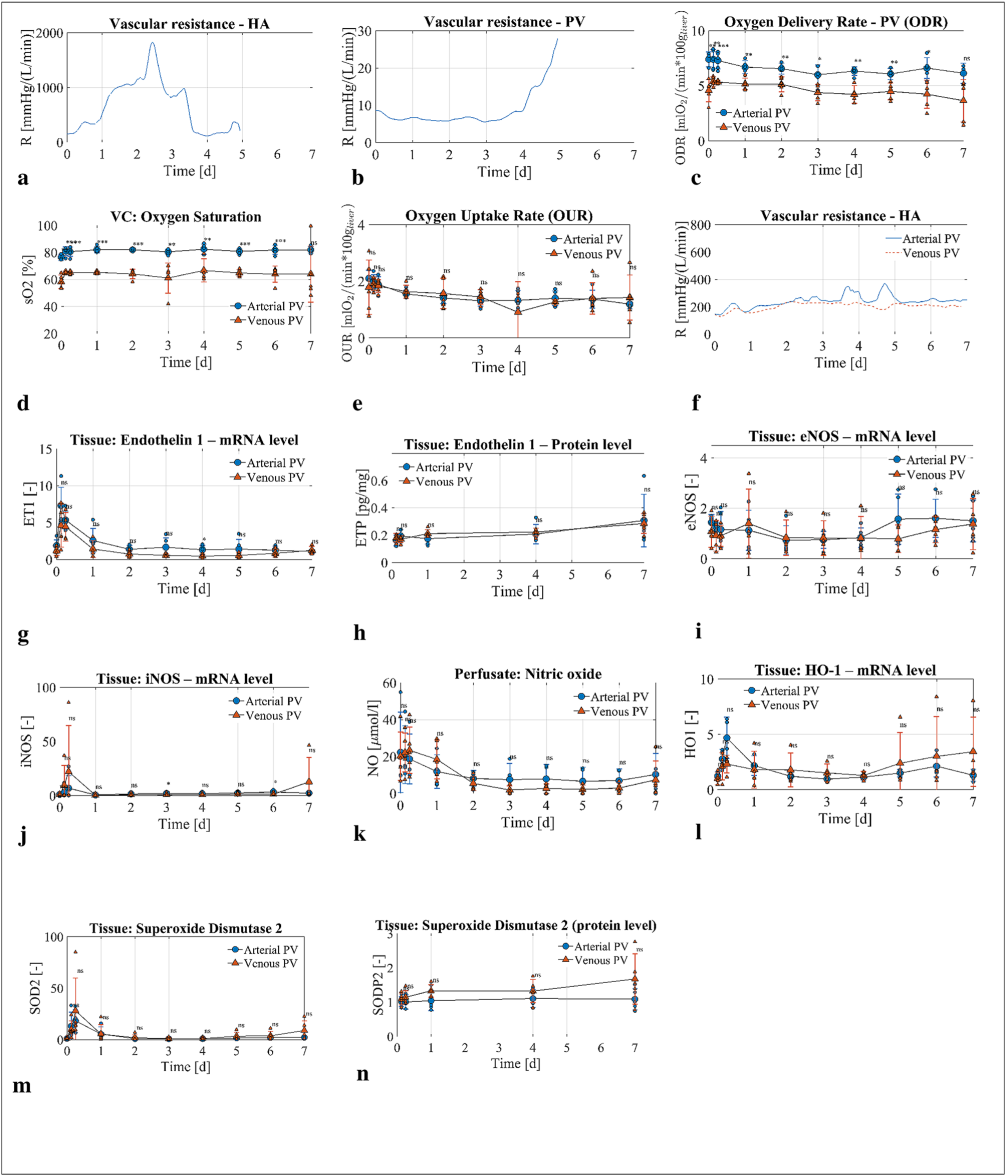
Oxygen delivery, vasculature resistance and patterns of vasoactive substance activation in experimental groups. Arterial PV group blue circles, n = 5 experiments, venous PV group red triangles, n = 5 experiments. (**a**,**b**) Representative experiment data of the HA and PV resistance from Group 0 without Nitroprussiat application. The increased HA resistance resulted in a decreased flow between perfusion day 1 and 3. From perfusion day 4, HA resistance decreased and was accompanied with increased PV resistance and absence of bile flow. (**c**) Oxygen delivery rate in the PV was higher with arterial PV compared to venous PV. (**d,e**) Oxygen saturation in the vena cava was higher in arterial PV group compared with venous PV group with no difference in oxygen uptake rate between groups during perfusion. (**f**) Resistance in arterial PV and venous PV groups controlled with Nitropurssiat. (**g**–**l**) Vasoactive substance release at mRNA and protein level without significant difference among experimental groups; (**g**) Endothelin-1 at mRNA level, (**h**) Endothelin-1 at protein level in tissue, (**i**) endothelial NO synthase (eNOS) and (**j**) inducible NO synthase (iNOS) at mRNA level, (**k**) NO level in perfusate, (**l**) heme oxygenase 1 at mRNA level. Superoxide dismutase (SOD) at mRNA (**m**) and protein (**n**) levels. P value * < 0.05, ** < 0.01, *** < 0.001. *ns* not significant.

Long-term liver perfusion was prevented by hemodilution and disturbed perfusate quality, including toxic uraemia (blood urea nitrogen > 100 mmol/l) and hypernatremia (sodium level > 200 mmol/l) in the absence of an integrated dialysis unit^5^. Thus, we integrated a dialysis unit to improve our protocol and machine development (Group 0). However, integration of dialysis alone was not sufficient to perfuse livers in a viable state for one week during initial experiments (Group 0). Specifically, while supplying the PV with arterial blood and applying dialysis, we observed increased HA resistance during perfusion, where the resistance increased compared with in vivo values (Fig. 2a). Livers stopped producing bile on perfusion day 3–6; the PV resistance increased at a similar time point (Fig. 2b). We terminated the experiments in this group prematurely on account of the cessation of bile flow cessation and portal hypertension (> 30 mmHg).

### Arterial oxygen saturation in the PV was associated with increased HA resistance

The observed increase in HA resistance was an intriguing finding. We hypothesized that control of HA resistance should enable long-term perfusion of viable livers. Therefore, we developed an automated algorithm to regulate HA resistance through the controlled supplementation of a vasodilator (Nitroprussiat). We suspected that hyperoxia contributed to the elevated arterial resistance, based on previous reports in experimental setting and humans^15–18^. In our protocol with arterial blood supplied to the PV (Group 1), high oxygen (DO_2_) was delivered to the liver through the PV (Fig. 2c). We observed vSO_2_ in liver output exceeding 80% and even reaching a level of close to 90% in some cases (Fig. 2d). To reduce the oxygen delivery to the liver (DO_2_), the PV was supplied with physiologic venous blood (Group 2; Fig. 1 b). To ensure sufficient oxygenation of the liver, the oxygen saturation in the vena cava (vSO_2_) was maintained at 65% based on current recommendations^19^. With venous oxygen content supplied to the PV (Group 2), significantly lower oxygen (DO_2_) was delivered to the liver compared with the same experiment using arterial blood in the PV (Group 1; Fig. 2c). Oxygen consumption (VO_2_) was not different between the two groups (Fig. 2e). The increased DO_2_ with arterial blood in the PV and no difference in VO_2_ resulted in increased vSO_2_ for Group 1 (arterial PV) as compared with Group 2 (venous PV; Fig. 2d). HA resistance was reduced with the supply of physiologic venous blood to the PV, as evidenced by the reduced demand for vasodilator at the same flow rate (Fig. 2f). The consumption of Nitroprussiat was reduced from 285 ± 162 ml/week in Group 1 to 41 ± 34 ml/week in Group 2 (p = 0.011), preventing hyperoxia mitigated HA resistance and enabling extended liver perfusion.

### What is the mechanism behind enhanced arterial vasoconstriction with arterial blood in PV?

To explore the underlying mechanism for the increased HA resistance with arterial blood in the PV, we investigated markers of vasoconstriction and vasodilation in the perfused livers. Specifically, we quantified the expression of Endothelin 1 (ET-1), a vasoconstrictor, and NO activity, a vasodilator. We observed no difference in ET-1 expression at the mRNA and protein levels between the two groups (Fig. 2g,h). To investigate if HA resistance was related to NO, we quantified NO activity in perfused livers from Groups 1 and 2. We observed no difference in the mRNA expression of endothelial nitric oxide synthase (eNOS) and inducible nitric oxide synthase (iNOS; Fig. 2i,j). Similarly, the concentration of NO in the perfusate did not differ between the two groups (Fig. 2k). However, the measured NO concentration in the perfusate of livers from both groups was lower than in vivo values. Note, the concentration measured in vivo corresponded to whole body levels, whereas only the liver was producing NO ex vivo, which might explain the lower NO concentration during perfusion. Moreover, NO was partially washed out by the dialysis unit. Further, we investigated the effect of NO on heme oxygenase 1 (HO1); HO1 mRNA expression was not different between the two groups, implying similar NO content in tissue (Fig. 2l). Since oxygen content was different in the tissue between groups with possible difference in superoxide levels, superoxide dismutase was measured. We did not find any difference at the mRNA and protein levels in superoxide dismutase between groups (Fig. 2 m, n).

### Venous blood delivery in the PV is safe and sufficient for 1 week liver perfusion

**Figure 3:**
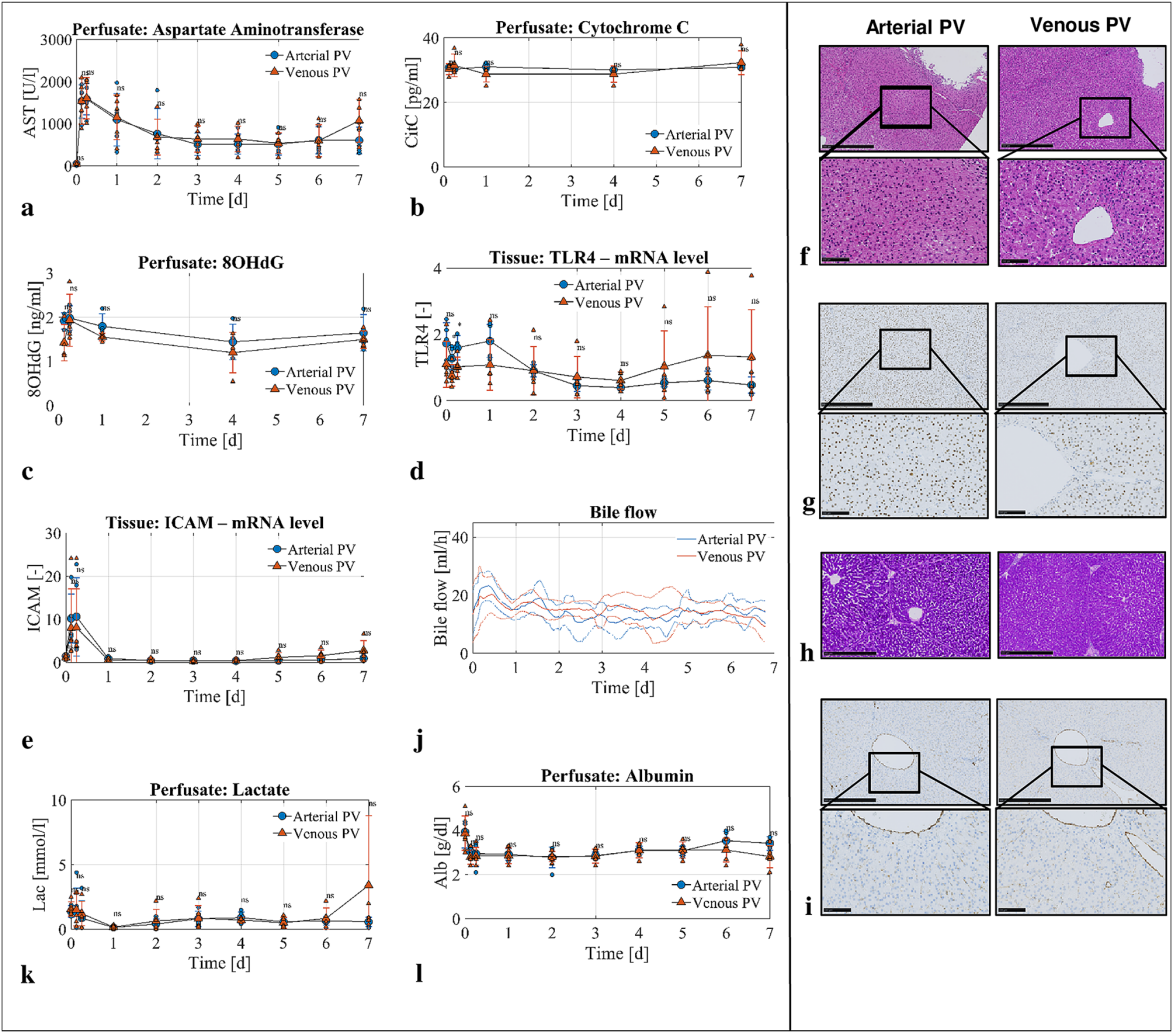
Injury markers and liver function in experimental groups. Arterial PV group blue circles, n = 5 experiments, venous PV group red triangles, n = 5 experiments. (**a**–**c**) Injury marker release in perfusate shown for AST (**a**), cytochrome c (**b**) and 8-OHdG (**c**). (**d**,**e**) Representative staining showing integrity on H&E staining (**g**) and preserved glycogen stores in PAS staining (**h**) after one week of perfusion. (**f**) Representative immunohistochemistry staining for Caspase 3 showing absence of relevant apoptosis on perfusion day 7. (**d**) Macrophages were not activated in both groups as shown with TLR4 at mRNA level. (**e**) Endothelial cell activation at mRNA level expressed with ICAM-1. (**i**) similarly to ICAM-1, von Willebrand Factor immunohistochemistry staining showed absence of relevant endothelial cell activation. (**j**) Bile flow was constantly present in both experimental groups for one week. (**k,l**) Livers cleared lactate (**k**) and maintained albumin level (l) in perfusate. P value * < 0.05, ** < 0.01, *** < 0.001. *ns* not significant.

To investigate the safety and efficacy of delivering venous blood in the PV, we analysed common injury parameters in both the perfusate and tissue as well as key liver functions. In the perfusate, we quantified aspartate aminotransferase (AST) as a marker of hepatocyte injury, Cytochrome C as a marker of mitochondrial injury, and 8-hydroxy-2′-deoxyguanosine (8-OHdG) as a marker of DNA damage. We observed no difference in AST, Cytochrome C, and 8-OhdG levels between the two groups (Fig. 3a–c). Next, we studied markers of tissue damage in the perfused liver tissue in each group. No difference was observed in mRNA expression of Toll-like Receptor 4 (TLR4), a marker of macrophage activation (Fig. 3d). Endothelial cells exhibited normal function based on Intracellular Adhesion Molecule 1 (ICAM-1) mRNA expression (Fig. 3e).

In addition, both groups demonstrated preserved liver structure based on histology with H&E staining, showing no relevant cell death and without caspase-3^+^cells (Fig. 3f,g). The livers also preserved glycogen in PAS staining, an indicator of liver health (Fig. 3h). Immunohistochemical staining of von Willebrand factor confirmed the observation of normal ICAM-1 expression (Fig. 3i). All livers in both groups, demonstrated normal liver function during sustained perfusion without any difference between groups. Livers produced bile (Fig. 3j), cleared lactate (Fig. 3k) and maintained albumin production (Fig. 3l). In total, these findings demonstrate that perfusion with venous blood in the PV is safe and sufficient during maching perfusion for one week.

## Discussion

This work explored the change in HA resistance depending on the oxygen content in the PV during ex vivo perfusion of porcine livers. The main findings of the study were, first, that arterial oxygen content in the PV delivers excessive oxygen to the liver. Second, hyperoxia causes HA vasoconstriction and prevents long-term perfusion. Third, the mechanism behind increased HA resistance with hyperoxia remains unclear. However, higher break down rates of NO due to increased oxygen delivery and direct oxygen sensing of endothelial cells might explain the increased resistance in HA with hyperoxia.

HA resistance regulation in vivo is complex, including extrinsic factors, such as neuro-humoral stimulation, which are missing in the ex vivo setting^7^. Thus, tight control over HA hemodynamics is required during machine perfusion. Our findings demonstrate that excessive oxygen delivery during ex vivo liver perfusion further compromises vasculature resistance regulation, which can be improved by physiologic oxygen delivery in the PV. The rationale behind non-physiologic arterial oxygen content in the PV at the start of our experimental campaign was based on existing perfusion protocols in the literature^8,9^. However, the liver has two hepatofugal vessels with venous blood in the PV and arterialization of the PV blood supply results in excess oxygen. Note, the observed hepatic venous blood oxygen saturation reaching 90% in arterial PV can be considered safe^10,20,21^.

To recapitulate the in vivo situation, we delivered venous blood in the PV as a next step in the perfusion protocol development. This resulted in the unexpected reduction in demand for vasodilator. Previous studies demonstrated that HA resistance increases in response to increased PV flow and vice versa. This phenomenon is coined in the literature as hepatic arterial buffer response (HABR)^7,22^. As this work shows, HA resistance changes also depending on oxygen content in the PV. It has been recognized that vascular endothelial cells regulate the vascular smooth muscle tone and can sense oxygen^23–25^. Endothelial cells have several oxygen sensing mechanisms, including heme oxygenase, oxygen-sensitive NADPH oxidases, and eNOS among others^25^. Hyperoxic vasoconstriction was first reported at least 100 years ago and confirmed consistently in in vivo studies^15,18,24,26^. The logical question that arises is how can hyperoxia in the PV reach the hepatic arteriole, which regulates blood flow in most organs. Although, the terminal branches of the hepatic arteriole and portal vein lie within an enclosed space of Mall, arterial and portal blood mix in sinusoids. The unique feature of the hepatic blood flow regulation is that sinusoidal endothelial cells together with hepatic stellate cells and vascular smooth muscle cells control the resistance in the hepatic vascular bed^27,28^. Indeed, vasoactive substances injected into the PV have similar access and effect on hepatic vascular bed compared to intra-arterial injection^7,29^.

Although our observation is consistent with the physiology, the mechanism behind these findings is still illusive with controversial results. Most studies about vascular resistance have explored the release of vasoactive substances ET-1 and NO^18,26,30^. ET-1 is a vasoconstrictor with the most long-lasting effect ever discovered^16,23,31^. Although ET-1 is a well-known vasoconstrictor, its production and release in vivo is still poorly understood^16,23^. Some studies claimed that high oxygen levels stimulate ET-1 production in vivo^16,23^. However, we could not confirm this hypothesis in our ex vivo setting.

Another vasoactive signaling molecule, NO, is involved in numerous physiological functions including vasodilation with a short half-life. Similar to ET-1, NO production was not different between the two groups. However, the applied Nitroprussiat is an NO donor and was infused at an increased rate with presence of excessive oxygen. The mechanism behind increased NO demand with excessive oxygen is possibly due to increased rates of metabolism; the steady-state NO level depends on its synthesis and metabolism and both are oxygen-dependent processes^32^. Although NO reacts with oxygen directly (autoxidation) at a very slow rate, the significant NO metabolism occurs through reactions with free radicals^32,33^. Superoxide is the most notable free radical in this context. Superoxide is a reactive oxygen ion and formed as a natural byproduct of oxidative metabolism^33^. Superoxide undergoes extremely rapid radical–radical reaction with unpaired electrons present in NO forming nitrates^30,33^. Its level is dependent on oxygen concentration^30,33,34^. Thus, we propose that increased NO break down rate in presence of excessive oxygen might underlie the increased need for NO donors by reacting with free radicals^30^. Owing to the very rapid metabolism rate, the detection/measurement of radicals was not possible in our setting. The increased NO need in excessive oxygen content was also shown consistently in studies with animals and tissues^30^.

Beyond increasing the HA resistance, excessive oxygen might have a negative effect in injured human livers, since they are more susceptible to hyperoxia as was proposed in a clinical liver transplant setting^11^. A group from Birmingham transplanted 12 initially discarded human livers. Of those, 6 were perfused with over oxygenation, including also arterial blood in the PV, while 6 were perfused with near-physiologic oxygen supply and venous blood in the PV. Although these authors did not note the clear difference in liver performance between high and low oxygen supply groups during ex vivo machine perfusion, the difference became apparent after transplantation. In this study, post-perfusion syndrome including primary graft non-function was linked to excessive oxygen during liver perfusion. Such a complication was not observed, when livers were perfused at near-physiologic oxygen tensions in the HA and venous blood in the PV^11^. This is the only study available in a clinical setting comparing the effect of excessive oxygen during ex vivo perfusion. The reported safety and feasibility of providing physiologic venous blood delivery in the PV during ex vivo perfusion with an optimized HA resistance regulation compared to non-physiologic arterial blood in the PV could support the application of such a perfusion loop in human livers grafts.

In the current manuscript we created partial oxygenation in the PV by mixing the blood (arterial and venous) directly in the PV line (Fig. 1 b). However, there are also other possibilities^8^. One option would be to use two oxygenators separately for HA and PV and then adjust gas flows individually for every oxygenator^35^. Using two oxygenators was upfront not an option in this project since the additional oxygenator increases the foreign surface area of the perfusion loop and thus, increases hemolysis. The oxygenator has the largest foreign surface area of all the system components. It also increases the machine costs and complexity, as it would require separate gas flow controllers (N_2_, O_2_, CO_2_)  for every oxygenator. Another option was to add a bypass from the oxygenator to the blood reservoir, which we tested initially in three experiments. With this approach, we observed a higher blood flow rate through the oxygenator to meet the oxygen demand. Moreover, we also observed a generally  higher hemoylsis rate (data not shown). Nonetheless, putting the bypass line directly from the HA to the PV line reduced the required flow rate and was suitable to maintain the desired physiologic oxygen saturation in the vena cava.

In conclusion, this work demonstrates that non-physiologic arterial oxygen content in the PV causes over supply of oxygen with consequent higher HA resistance. Physiologic venous blood in the PV during ex vivo liver perfusion is preferred as it improves the resistance control in the HA. Although the mechanism behind increased HA resistance by over oxygenation is not entirely clear in our system, we propose direct oxygen sensing of sinusoidal endothelial cells and/or increased NO break down rate with hyperoxia as possible explanations.
